# FishNet: an online database of zebrafish anatomy

**DOI:** 10.1186/1741-7007-5-34

**Published:** 2007-08-17

**Authors:** Robert J Bryson-Richardson, Silke Berger, Thomas F Schilling, Thomas E Hall, Nicholas J Cole, Abigail J Gibson, James Sharpe, Peter D Currie

**Affiliations:** 1 The Victor Chang Cardiac Research Institute, 384 Victoria Street, Darlinghurst, Sydney, 2010, Australia; 2 Department of Developmental and Cell Biology, School of Biological Sciences, University of California, Irvine, 5205 BioSci II (McGaugh Hall), Irvine, CA 92697-2300, USA; 3 Centre for Genomic Regulation, C/Dr. Aiguader 88, 08003 Barcelona, Spain; 4 School of Biotechnology & Biomolecular Sciences, University of New South Wales, Sydney, NSW 2052, Australia; 5 St Vincent's Clinical School, University of New South Wales, Sydney, NSW 2052, Australia

## Abstract

**Background:**

Over the last two decades, zebrafish have been established as a genetically versatile model system for investigating many different aspects of vertebrate developmental biology. With the credentials of zebrafish as a developmental model now well recognized, the emerging new opportunity is the wider application of zebrafish biology to aspects of human disease modelling. This rapidly increasing use of zebrafish as a model for human disease has necessarily generated interest in the anatomy of later developmental phases such as the larval, juvenile, and adult stages, during which many of the key aspects of organ morphogenesis and maturation take place. Anatomical resources and references that encompass these stages are non-existent in zebrafish and there is therefore an urgent need to understand how different organ systems and anatomical structures develop throughout the life of the fish.

**Results:**

To overcome this deficit we have utilized the technique of optical projection tomography to produce three-dimensional (3D) models of larval fish. In order to view and display these models we have created FishNet http://www.fishnet.org.au, an interactive reference of zebrafish anatomy spanning the range of zebrafish development from 24 h until adulthood.

**Conclusion:**

FishNet contains more than 36 000 images of larval zebrafish, with more than 1 500 of these being annotated. The 3D models can be manipulated on screen or virtually sectioned. This resource represents the first complete embryo to adult atlas for any species in 3D.

## Background

Zebrafish possess a number of attributes that have facilitated their uptake as a developmental model system. Zebrafish uniquely combine embryological manipulability, optical clarity of the early embryo and larvae (allowing simple visualization of cell biological events directly *in vivo*) and the ability to apply invertebrate-style forward genetics to questions of vertebrate development. More recently, research has extended into later aspects of zebrafish development and adulthood, examining aspects of organogenesis and tissue maintenance. Many of the same strengths that made zebrafish a superior model for the study of development also complement those of existing mammalian disease models. Thus a huge variety of human conditions are now being modeled in zebrafish, ranging from drug and alcohol addiction to cancer [1-8].

The usefulness of any model organism is limited by the available accurate anatomical information for that system. At present, there is a lack of a detailed anatomical reference for zebrafish. While the earliest stages of zebrafish development have been intensively studied over the last two decades, and an embryological staging series described [9], comparatively little is known about the development of larval, juvenile and adult anatomy. Whilst the vascular anatomy of the embryonic fish [10], the anatomy of the embryonic and adult brain [11,12], and heart [13] have been elegantly described, there is a need for a detailed record of zebrafish development that spans the entire life history of the organism and presents its anatomy.

Traditionally, our understanding of anatomy has relied on the interpretation of two-dimensional (2D) images extrapolated into three-dimensions (3D). Data recorded in this manner are difficult to reinterpret, with the original anatomical representation conveying only one aspect of the data. Such data are also unable to be further interrogated in the context of the sample as a whole. If by contrast, data is captured for the entire sample in 3D it can be viewed, digitally sectioned, and interrogated in any way the researcher desires. A holistic understanding of the anatomy of an organism is critical to dissecting the development and function of different organs and tissues in space and time. We have therefore generated a database of zebrafish anatomy charting development at multiple time points in 3D.

While a number of different methods exist for the capture and generation of 3D images; such as the reconstruction of serial physical sections [14-17] from serial optical sections using confocal microscopy [18], or from magnetic resonance imaging (MRI); these methods all possess specific limitations that prevent their application to a 3D atlas of zebrafish development. MRI has not been adapted for use in the zebrafish due to the small size of its embryo and the current lack of resolution of this technique. Furthermore, the specialized equipment required for the application of MRI imaging has prevented its uptake for routine laboratory imaging. In the zebrafish, the 3D analysis undertaken so far has largely depended upon confocal imaging [10] or to a much lesser extent serial sectioning. However, neither of these techniques is currently suitable for the generation of a 3D atlas of zebrafish development. Conventional confocal microscopy is limited by the depth of tissue that can be examined, restricting its use to the earliest stages of embryonic development. While deeper penetration can be achieved using multi-photon imaging, this procedure is still insufficient to access all stages of zebrafish development, and all forms of confocal imaging rely exclusively on the use of fluorescent probes. In addition, 3D models created by the alternative method of serial sectioning are highly time consuming to generate and physical distortion of the sample can often occur during sectioning. Models from serial sections often have high quality images in the plane of section, however, difficulties in aligning the serial sections can lead to poor quality images when viewed in other planes.

We have therefore decided to adapt the method of optical projection tomography (OPT; [19,20]) to create a digital full life atlas of zebrafish development. OPT uses projection images of the sample to reconstruct a 3D model of the sample (demonstrated in Additional file 1), a process that overcomes many of the shortcomings of the other methods listed above. OPT can be used to create 3D models over a wide range of sample sizes, from 1–10 mm, covering the range of zebrafish development from the early embryo until adulthood. OPT can utilize both fluorescent or conventional brightfield labeling techniques, although prior to this publication its application has been limited to the generation of grayscale 3D models. As the OPT technique is non destructive there is no distortion of the sample similar to that induced by serial sectioning and consequently a smooth 3D model can be created, resulting in high quality images in all planes, not just that of the plane of section. Furthermore, the use of a single sample to generate all of the sections in any plane allows the user to examine the same structure at multiple angles, giving a clear representation of the 3D organization of the sample.

We have utilized OPT to create the first 3D lifespan atlas for any species, in the process generating 18 3D models spanning the entire period from embryo to adulthood. In order to make this data available to the research community we have created a specifically designed web interface [21] to allow the data to be viewed and manipulated. This resource contains more than 36 000 digital section images and the complete 3D models can be downloaded to allow interrogation in any plane the researcher desires. To further enhance the usefulness of this resource we have annotated more than 1 500 of the sections throughout the 3D models we have created with apparent anatomical structures. As such, FishNet represents the first complete embryo to adult atlas in 3D for any species.

## Results and Discussion

### Generating 3D models of zebrafish development

We collected samples representing the entire development of the zebrafish from early embryo until adulthood. Samples were collected at 24, 48, 72, 96, and 120 h post-fertilization (hpf). After the 120 hpf time point embryos were collected at specific total lengths (jaw to tip of tail), as length was more representative of developmental stage than age in later development, at each 1 mm stage from 5–17 mm. Using the OPT method we have developed for zebrafish [19] we generated a series of 3D models charting the development of the zebrafish, *Danio rerio*, from embryo to adult.

**Figure 1:**
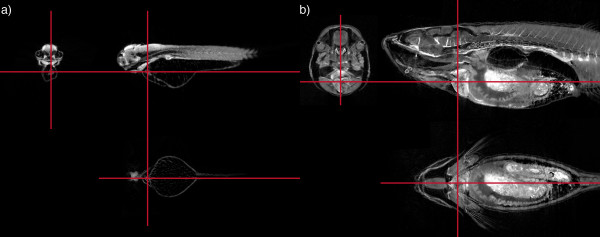
**Three virtual sections through 3D models of a 72 hpf embryo (A) and 16 mm fish (B), showing the heart**. Multiple virtual sections can be cut from the same 3D reconstructed model. The red lines show the positions of the sections relative to each other. Because of the 3D nature of the data individual structures can be easily located and viewed from multiple angles. The development of an individual organ or structure can be followed through the series of 3D models. In this case the sections are centred on the cardiac ventricle.

Each of these models can be virtually sectioned in any plane providing the ability to study the development of specific tissue or organs at multiple time points, encompassing the complete lifespan of the zebrafish. An example of this is shown in Figure 1, where multiple section images created from the same 3D model examining the heart at both 72 hpf and 16 mm stages are displayed, demonstrating the representation of 3D organization of an individual structure at specific stages of development.

### Analysis and 3D display

**Figure 2:**
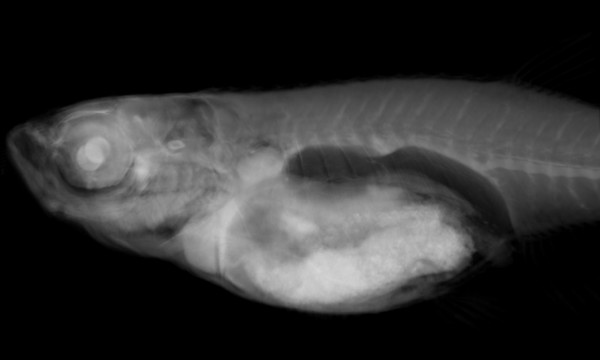
**Volume rendering of a 15 mm fish**. Volume rendering creates a 2D image that gives the impression of a 3D object. By reducing the opacity of the rendering internal structures can be distinguished.

Collecting 3D data, particularly using a method such as OPT that does not require alignment of physical sections, allows smooth 3D renderings of the samples to be created. Renderings are a powerful way to represent the 3D organization of the sample in a 2D image. By reducing the opacity of the model volume renderings can generate images containing all of the data. By increasing the opacity of each pixel with intensity we can create a rendering that is highly representative of the original sample (Figure 2). These volume renderings allow more natural looking surfaces with smooth edges compared to other types of threshold renderings, and we have created volume renderings of all of the models in our dataset. These renderings can be freely manipulated on screen, changing orientation, position, and magnification. Thus, an individual structure can be viewed at any angle.

### FishNet: an interface for the database

**Figure 3:**
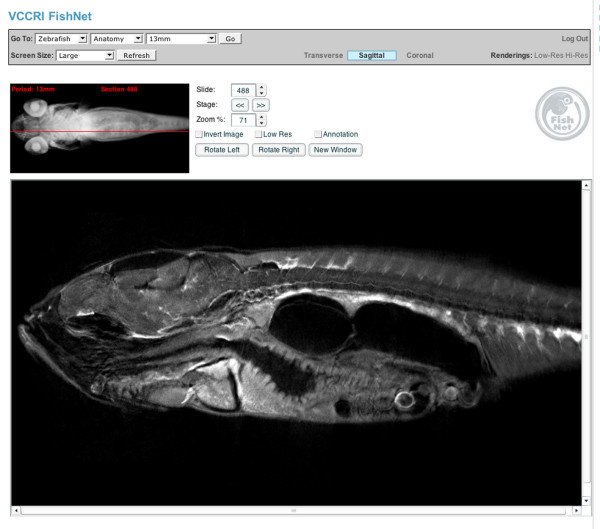
**The FishNet interface**. Users can determine the plane of section and the position of the section by looking at the volume rendered image top left of the screen. Sections can be selected by dragging the red bar on the volume rendered image, using the cursor keys or by typing in the number of the section they wish to view.

Having created models covering the complete range of development we designed a novel web interface, FishNet, to allow easy online access to the full dataset. The tools we have implemented allow the 3D models to be virtually sectioned and manipulated within the browser. To navigate through the 3D data we have generated, the user is prompted with a volume rendering of the entire sample to which individual sections of the model are linked, with the position of the section within the 3D model being highlighted on the rendered image (Figure 3) The user is then able to move through the sample in any of the three standard section views; transverse, sagittal, or coronal. The three standard section planes are available on the web interface and the complete 3D models are available for download. These complete 3D models can then be sectioned in any desired plane. FishNet contains all of the section images of zebrafish development we have generated: over 36 000 in total. To facilitate use by users without a fast Internet connection, we have reduced size versions of every section image and users can choose to browse the reduced size versions before selecting a higher resolution copy to examine. In this way, the vast number of images can be easily browsed in an intuitive manner on the wide range of operating systems and Internet connections used by researchers.

The user can change the magnification of the viewed image to browse either the entire section or to closely examine a selected region. The image can also be dragged on screen to centre on a region of interest. Once a particular section of interest has been identified, the user can also open this in a separate new window while continuing to browse the database, facilitating comparison between stages and views.

To allow users of the website to view the 3D renderings of the models in an Internet browser we have generated rendered images of the models viewed at 162 different angles. These pre-computed renderings are then used to create a QuickTime virtual rendering (QTVR) for each model. These QTVRs can be viewed in a browser window and the sample moved in 3D using the mouse or cursor keys. As the renderings are pre-computed, once loaded, the QTVR can be manipulated instantly and the user can rotate the model freely. To ensure access to all users, we have also included smaller versions of the QTVR with 18 pre-computed views compared to 162 in the standard versions; these smaller versions will be more accessible to those with limited Internet connections.

### An anatomical reference for the zebrafish

**Figure 4:**
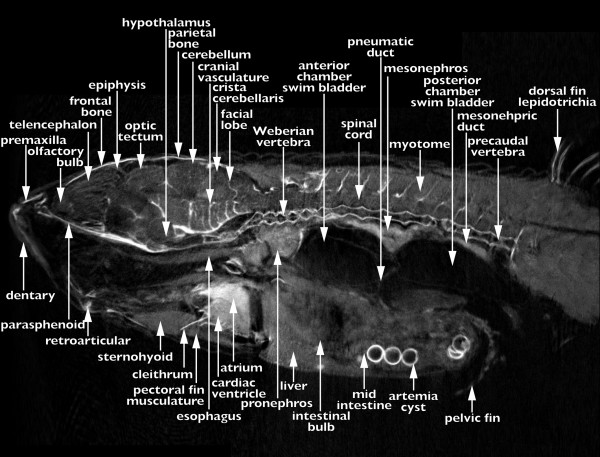
**An annotated reference of zebrafish development**. One of more than 1 500 annotated sections present within the FishNet anatomy atlas. This section is of an adult (17 mm) fish in sagittal view at the midline.

To allow use of the database as a reference and educational resource we have annotated at least 90 sections for each stage, 30 in each view; transverse, sagittal, or coronal; a total of more than 1 500 annotated images. We have provided an example of a typical annotated section in Figure 4. All of the annotations use approved zebrafish nomenclature [22]. Users can select to browse the data looking at every section or only those that have been annotated. When browsing un-annotated sections the user can move to the closest annotated section by selecting one of the buttons on screen.

### Color OPT

A major limitation of the current OPT system is that it is only able to capture grayscale images. Although fluorescent OPT models can be pseudo-colored following reconstruction, to represent the color of the fluorescent probe, transmission or brightfield OPT has so far been limited to producing grayscale models. This has prevented its use with many of the commonly used histological stains that rely on color differences to provide information about the intensity and levels of a signal and the use of multiple color stains together.

**Figure 5:**
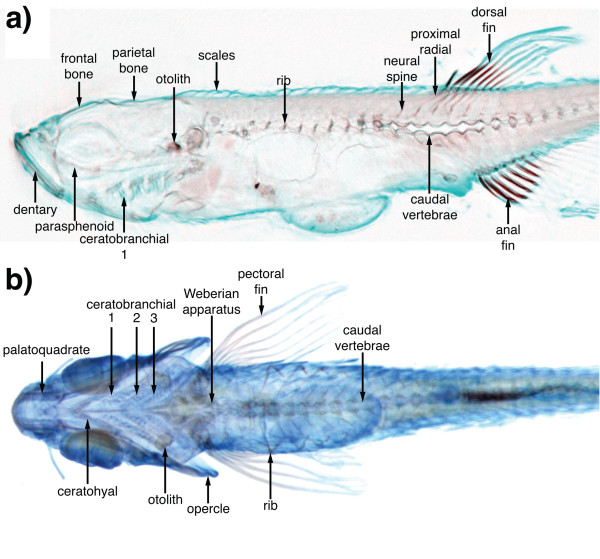
**cOPT reconstruction**. A 10 mm fish is shown with Alcian blue and alizarin red staining the cartilage and bone respectively. The three separate reconstructions relating to the red, green, and blue channels are combined to form a single cOPT image allowing a combination of brightfield stains to be used. (A) Sagittal section. (B) Color volume rendering showing the complete developing skeleton of the 10 mm fish.

We have modified and improved the OPT technique such that we are now, for the first time, able to scan multi-color samples and faithfully reconstruct these images in 3D, and have termed this technique cOPT. At each angle of rotation three separate images are captured through an LCD filter alternating between red, green, and blue. Each color channel is then separately reconstructed before merging to reconstruct a complete color model. This allows multiple stains to be used in combination within a single sample. Such an ability represents a large step forward for the versatility of the OPT system. In order to demonstrate the ability to capture color 3D images we scanned, using cOPT, bone and cartilage development using alizarin red and Alcian blue stains, respectively, and created 3D models of the developing skeleton. We provide examples of these reconstructions in Figure 5. This model of the skeleton at 10 mm represents the start of an ongoing effort to record organogenesis and the development of individual structures in 3D.

## Conclusion

A holistic understanding of the anatomy of an organism is critical to dissect the development and function of different organs and tissues. Information about the spatio-temporal patterning of an anatomical structure can be invaluable in understanding its development and effect on surrounding structures. The most complete mechanism for generating this understanding is to represent development in four dimensions. Using OPT we have created an online database recording development in 3D at a complete range of stages from early embryo to adulthood.

Given the greater clarity with which anatomical information can be relayed in 3D it is clear that the future for data representation in whole organismal context is 3D. The relative ease of data capture by OPT, the establishment of rendering and display tools to display this captured data, suggests that OPT might become one of a standard set of tools for the analysis of zebrafish anatomy, gene, and protein expression. Recently, further improvements to OPT have been described that improve the resolution obtained through use of a frequency space filter to remove out of focus information prior to reconstruction [23]. As these improvements and others are incorporated into OPT reconstructions, the quality and resolution of the images will continue to improve. The ability to create 3D models of color samples using cOPT greatly increases the number and combination of stains that can be analyzed by this method, and also allows the results to be presented in a more recognizable way to the user.

In order for the data to be of use it is vital that it is presented in an accessible and intuitive manner. Establishing user friendly Internet-based tools to allow the virtual sectioning of the 3D models and the manipulation of 3D models ensures the data is available to all without the need for specialist equipment or software. The establishment of this reference for zebrafish development and anatomy will facilitate research using this model system, in particular research into later stages of development and adulthood, which are of special interest for the modeling of human disease. The annotation of a large and representative fraction of the database makes FishNet a valuable resource not only for zebrafish researchers but also to those in other disciplines who wish to further examine results in fish or undertake comparative analyses. FishNet will also be of great use as an educational resource.

Our development of FishNet is the first step towards developing a fully integrated repository for 3D data for anatomy and development in the zebrafish. We plan to continue to build this resource adding models following organogenesis through the labeling of individual organs at multiple time points and hope that in the future FishNet will serve as a repository for 3D gene expression information. As more data are added to FishNet we will also continue to develop tools for the interrogation of this data set to make use of this 3D resource.

## Methods

### Sample preparation

Zebrafish were obtained from wild-type or golden mutant lines. Samples were fixed in 4% paraformaldehyde overnight at 4°C, and then washed with PBS containing 0.1% Tween 20. Samples were used immediately or progressed through a methanol series for storage in 100% methanol at -20°C. Pigmented embryos were bleached in a 5% formamide, 10% H_2_O_2_, 0.5 × SSC solution, following dehydration and re-hydration in a methanol series. Samples were embedded in a 1.5% low melting point agarose gel and attached to a metal mount. The mounted samples were then cleared through a series of 25%. 50%, 75%, 2 × 100% methanol prior to immersion in a 2:1 benzyl alcohol:benyzl benzoate solution.

### Optical projection tomography

The projection images were captured using a Retiga 1300Exi ccd camera (QImaging; 19535 56th Avenue, Suite 101, Surrey, BC, Canada. V3S 6K3) mounted on a Leica MZFLIII stereo-fluorescent microscope with HBO 100 fluorescent lamp source and a GFP1 filter for auto-fluorescent capture, and a LCD RGB filter (QImaging) for color samples. The samples were mounted in the 'Edinburgh OPT Scanner', and a series of 400 images captured over a 360° rotation. Images were captured at a 1360 × 1024 pixel resolution. A detailed description of the equipment required and method for preparation of samples and subsequent 3D reconstruction is given in Bryson-Richardson and Currie [19].

### 3D Rendering

The 3D models were converted to .slc format files and rendered using the visualization toolkit either for direct display on screen, or to produce a series of rendered images of the rotating sample. These were then subsequently used to create QuickTime virtual renderings (QTVR rendering) using The VR Worx (VR Toolbox Inc; P.O. Box 111419, Pittsburgh, PA 15238 USA).

### Color OPT reconstruction

Each color channel was reconstructed individually. The complete 3D models were then examined to see the range of grey values they contained. All three images were then converted to 8-bit using the same limits as the channel with the greatest range of values. Section images and volume renderings were created for each model individually and merged using ImageMagick (on-line resource [24]) convert to produce a color image.

## Authors' contributions

RJBR developed OPT for use in zebrafish, conceived and designed FishNet, generated 3D models, annotated the anatomy, and drafted the manuscript. SB generated 3D models. TFS, TEH, NJC, and AJG assisted in the anatomical annotation. JS developed the OPT system. PDC assisted in the design of FishNet, the annotation of the anatomy and the drafting of the manuscript.

## Supplementary Material

Additional file 1 **Principles of tomographic reconstruction**. The sample (blue) has been cleared to allow light to pass through. Light passes through the tissue depending on the depth and density of the tissue. The image collected of the light passing through the sample is a projection image, as used in OPT. If projection images are collected at multiple angles as the sample is rotated the shape of the object can be reconstructed. On the right-hand side of the image the projection images are back projected and as more angles are added they create the shape of the original sample. In this example, 19 different angles are used. Back projection is the simplest method of reconstruction and many of the evident artefacts are removed using the algebraic methods utilized for OPT reconstruction. Click here for file
